# Transcriptomic datasets of cancer patients treated with immune-checkpoint inhibitors: a systematic review

**DOI:** 10.1186/s12967-022-03409-4

**Published:** 2022-05-31

**Authors:** Szonja Anna Kovács, Balázs Győrffy

**Affiliations:** 1 Department of Bioinformatics, Semmelweis University, Tűzoltó utca 7-9, 1094 Budapest, Hungary; 2 Research Centre for Natural Sciences, Oncology Biomarker Research Group, Institute of Enzymology, Eötvös Loránd Research Network, Magyar Tudósok körútja 2, 1117 Budapest, Hungary

**Keywords:** PD-1, PD-L1, CTLA-4, Survival, Response, Clinical data, Gene expression

## Abstract

The availability of immune-checkpoint inhibitors (ICI) in the last decade has resulted in a paradigm shift in certain areas of oncology. Patients can be treated either by a monotherapy of anti-CTLA-4 (tremelimumab or ipilimumab), anti-PD-1 (nivolumab or pembrolizumab), or anti-PD-L1 (avelumab or atezolizumab or durvalumab) or as combination therapy of anti-CTLA-4 and anti-PD-1. To maximize the clinical treatment benefit of cancer immunotherapy, the prediction of the actual immune response by the identification and application of clinically useful biomarkers will be required. Whole transcriptomic datasets of patients with ICI treatment could provide the basis for large-scale discovery and ranking of such potential biomarker candidates. In this review, we summarize currently available transcriptomic data from different biological sources (whole blood, fresh-frozen tissue, FFPE) obtained by different methods (microarray, RNA-Seq, RT-qPCR). We directly include only results from clinical trials and other investigations where an ICI treatment was administered. The available datasets are grouped based on the administered treatment and we also summarize the most important results in the individual cohorts. We discuss the limitations and shortcomings of the available datasets. Finally, a subset of animal studies is reviewed to provide an overview of potential in vivo ICI investigations. Our review can provide a swift reference for researchers aiming to find the most suitable study for their investigation, thus saving a significant amount of time.

## Immune-checkpoint inhibitors (ICIs)

In physiological conditions, immune checkpoints are crucial to prevent exaggerated inflammation, which would otherwise cause serious damage to the tissues. Thus, these ‘brakes’ are essential for preventing autoimmunity. However, cancer cells can also acquire the ability to suppress the immune response and evade recognition and elimination by immune cells. Stimulation of T-cell mediated innate (via CD8 + cytotoxic T-cells) and adaptive (via CD4 + helper T-cells) immune response is a major aspect of immuno-oncology.

**Fig. 1:**
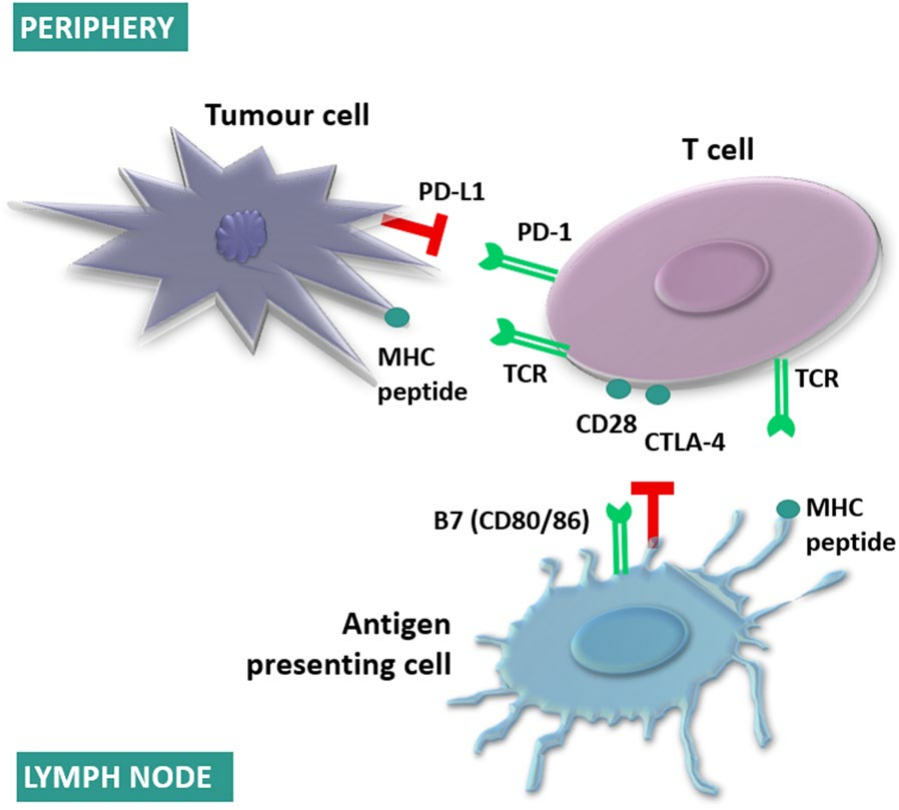
Activatory (green) and inhibitory (red) signals in immune-checkpoint inhibition

Cytotoxic T lymphocyte-associated protein 4 (CTLA-4, also called CD152) was the first checkpoint inhibitor to be clinically targeted [1, 2]. CTLA-4 is an intracellular protein constitutively expressed at a lower level in resting T cells [3]. When a T cell receptor (TCR) binds an antigen, and costimulatory signals of cluster of differentiation 28 (CD28) also arise, CTLA-4 is translocated to the cell membrane where it competes with CD28 for binding to one of its two ligands: B7-1 (CD80) and/or B7-2 (CD86) (Fig. 1). While CD28 is a positive costimulator of CD80/86, CTLA-4 mediates a negative, inhibitory signal upon binding (e.g. by reducing CD4 + helper T cell activity) [3, 4].

Programmed cell death 1 (PD-1) receptor is a transmembrane protein expressed on T cells (and also B cells, NK cells, myeloid suppressor dendritic cells (DCs) [5]). PD-1 controls T cell activation and tolerance and reduces inflammation. PD-1 has two ligands: programmed cell death ligand 1 (PD-L1, also known as CD274 or B7-H1) and programmed cell death ligand 2 (PD-L2, also known as CD273 or B7-DC) (Fig. 1). PD-L1 can be detected on many somatic cells, including non-hematopoietic tissue cells (e.g. endothelial and epithelial cells) and hematopoietic cells (e.g. T cells, B cells, macrophages, DCs, mast cells), while PD-L2 is expressed mainly by DCs, macrophages, and mast cells. Both PD-L1 and PD-L2 can also be found on tumor cells and stromal cells (e.g. fibroblasts, immune cells, endothelial cells), contributing to T cell exhaustion, immunosuppression, induction of regulatory Tregs, and decreased T cell cytotoxic activity [6, 7].

## Anti-CTLA-4 antibodies

Ipilimumab is an IgG1 monoclonal antibody (mAb) that was approved by the United States Food and Drug Administration (US FDA) for the treatment of metastatic melanoma in 2011 [8, 9]. In the last 10 years, further indications were approved in different subsets of melanoma either as a monotherapy or as a combination therapy with nivolumab, like in B-Raf Proto-Oncogene (BRAF) V600 wild-type unresectable or metastatic melanoma [10]. The ipilimumab plus nivolumab combination was also approved for advanced renal cell carcinoma [11], microsatellite instability-high/mismatch repair-deficient (MSI-H/dMMR) metastatic colorectal carcinoma [12], hepatocellular carcinoma [13], and metastatic non-small cell lung cancer (NSCLC) in case the tumor cells express PD-L1 (≥ 1%) [14] or regardless of PD-L1 expression [15], and for unresectable malignant pleural mesothelioma [16]. Although the mAb tremelimumab (CP-675,206; formerly ticilimumab) is not approved by the FDA, it received an Orphan Drug Designation for the treatment of malignant mesothelioma after the results of the DETERMINE trial had come out. Malignant mesothelioma is an asbestos-related rare but extremely lethal tumor of the mesothelial surfaces of the pleura and peritoneum [17]. Because of this, tremelimumab was granted an Orphan Drug Designation only. Though this does not guarantee the efficacy and safety of a drug, these applications could lead to future approval processes.

## Anti-PD-1 antibodies

Pembrolizumab is a humanized mAb against PD-1 approved by the FDA in 2014 after the KEYNOTE-001 clinical trial supported its efficiency in patients with unresectable or metastatic melanoma [18] and patients with NSCLC [19]. Further approved indications of pembrolizumab include NSCLCs with a positive proportion of PD-L1 over 1%, recurrent or metastatic squamous cell carcinoma of the head and neck (HNSCC) [20], recurrent or metastatic cervical cancer, locally advanced or metastatic urothelial carcinoma [21], locally advanced or metastatic squamous cell carcinoma of the esophagus [22], locally advanced or metastatic gastric or gastroesophageal junction carcinoma with a PD-L1 expression score of CPS ≥ 1 (combined proportion score) [23], and for the treatment of locally recurrent unresectable or metastatic triple-negative breast cancer (TNBC) with a CPS ≥ 10 [24]. MSI-H or dMMR are also indications for pembrolizumab therapy regardless of tumor origin [25]. According to the results of the KEYNOTE-158 trial, high tumor mutational burden (TMB-H) can also be used as a predictive biomarker [26].

Nivolumab is a mAb (IgG4) approved by the FDA in 2014 based on the results of the CheckMate-037 trial where unresectable, metastatic melanoma patients progressing after ipilimumab treatment were investigated [27]. As of today, further indications are also accepted including metastatic NSCLC with or after chemotherapy, advanced renal cell carcinoma, Hodgkin’s lymphoma, and metastatic HNSCC [5]. The reliability of PD-L1 status alone as a predictive biomarker for nivolumab response is still under debate as contradictory results have been published in retrospective studies of NSCLC, urothelial carcinoma, melanoma, and esophageal cancer [5]. The CheckMate-142 phase II study also validated nivolumab for the treatment of MSI-H/dMMR metastatic colorectal cancer [28].

Advanced or metastatic cutaneous squamous cell carcinoma (CSCC) is the second most common skin cancer and is widely known for its high tumor mutational burden (caused by ultraviolet radiation, age, and immunosuppression). In this context, a reasonable step was to develop the new anti-PD1 mAb (IgG4) cemiplimab-rwlc which was approved for the systematic treatment of CSCC in 2018 [29]. More recent studies have suggested the benefits of cemiplimab treatment in recurrent or metastatic cervical cancer [30], and advanced or metastatic NSCLC [31].

In China, sintilimab [32], camrelizumab [33], and tislelizumab [34] are approved for the treatment of classical Hodgkin’s lymphoma and are currently under investigation in the US by the FDA for a different types of cancers. Sintilimab (ORIENT-11) [32, 35] and camrelizumab [33] are investigated in advanced or metastatic non-squamous NSCLC, and camrelizumab is evaluated in nasopharyngeal cancer (CAPTAIN-1st) [36], hepatocellular carcinoma, B cell lymphoma, esophageal squamous cell carcinoma, and gastric/gastroesophageal junction cancer as well [33]. In 2021, the FDA approval of tislelizumab was announced for the treatment of unresectable recurrent locally advanced or metastatic esophageal squamous cell carcinoma (ESCC) based on the results from the RATIONALE-302 trial. Another clinical study is ongoing for squamous NSCLC (NCT03594747). Toripalimab was first approved in China to treat unresectable or metastatic melanoma (POLARIS-01) [37, 38], and promising effects have been reported in nasopharyngeal carcinoma as well (POLARIS-02) [39].

## Anti-PD-L1 antibodies

Atezolizumab is an anti-PD-L1 mAb (IgG1) available for various types of cancer. It was approved in 2016 for the treatment of advanced or metastatic urothelial carcinoma [40], since then indications widened and now include advanced melanoma, NSCLC (if PD-L1 expression is over 50% of tumor cells or over 10% of tumor-infiltrating immune cells), TNBC (if tumor-infiltrating immune cells ≥ 1%), renal cell carcinoma, HNSCC, colorectal carcinoma, hepatocellular carcinoma, and gastric carcinoma [5]. The IgG1 mAb avelumab was approved in 2017, 1 year after atezolizumab, for metastatic Merkel cell carcinoma (phase II JAVELIN Merkel 200 trial) which is a rare but immensely aggressive type of skin cancer [41]. Later, an indication of avelumab was approved in urothelial carcinoma and renal cell carcinoma (phase Ib JAVELIN Solid Tumor trial) [42]. PD-L1 positivity status was not predictive in any of these studies. Durvalumab is a mAb (IgG1κ) approved in 2017 for advanced or metastatic urothelial carcinoma—as previously, these patients showed benefits regardless of PD-L1 status [43]. A year later, a new indication was approved for advanced stage SCLC patients [44]. Currently, there is no clear evidence of improved benefits in the treatment of other cancers though further investigations are currently ongoing.

## Adjuvant or neoadjuvant administration of ICIs

Several clinical trials finished and still ongoing evaluate the optimal administration strategy of checkpoint-inhibitors including assessment monotherapy and combination therapy and evaluation of treatment timing including adjuvant, neoadjuvant, or the combination of adjuvant and neoadjuvant therapy. Other studies check the clinical benefits with and without simultaneous chemotherapy and/or radiotherapy. There is no universally accepted protocol for all patients regarding the optimal therapy, which can also vary due to tumor type, stage, mutational status, medical history, etc.

In (locally) advanced esophageal squamous cell carcinoma, concomitant or sequential administration of neoadjuvant nivolumab in combination with chemotherapy and surgery has shown mixed results according to the NCT03914443 trial [45]. The NCT02743494 trial studied the combination of chemoradiotherapy, surgery, and adjuvant nivolumab (primary outcome is disease-free survival, phase III), and found out that disease-free survival was longer in patients who had neoadjuvant chemoradiotherapy combined with adjuvant nivolumab [46].

For advanced (stage III-IV) melanoma, multiple trials have investigated the effects of neoadjuvant immunotherapy including NCT02437279 (ipilimumab and nivolumab, either adjuvant or neoadjuvant and adjuvant, phase I), NCT02977052 (neoadjuvant ipilimumab and nivolumab, phase II), NCT02519322 (neoadjuvant nivolumab with or without ipilimumab or relatlimab, phase II), NCT02434354 (neoadjuvant pembrolizumab, phase I), and NCT01608594 (neoadjuvant ipilimumab and high-dose interferon alfa-2b (INF-α2b), phase I). These studies concluded that neoadjuvant administration of ICIs is more favorable in metastatic melanoma, and leads to prolonged survival and higher pathological response rates [47–49]. Adjuvant settings are still being studied by multiple trials [50].

There are some ongoing phase III clinical trials assessing the value of adjuvant ICIs in stage I-III NSCLC including pembrolizumab after resection with or without chemotherapy (NCT02504372), atezolizumab after resection, and adjuvant chemotherapy (NCT02486718), nivolumab after surgery and chemotherapy (NCT02595944), and adjuvant administration of durvalumab (NCT02273375). Treatment with neoadjuvant and adjuvant atezolizumab in stage I-III NSCLC (NCT02927301) showed a 19% major pathological response (MPR) rate in a phase II study, while neoadjuvant nivolumab with chemotherapy delivered 83% MPR and 71% pathological complete response in stage III NSCLC (NCT03081689). Many trials regarding neoadjuvant ICI safety and efficacy are still ongoing [51].

## Transcriptomic datasets

Currently used biomarkers, such as PD-L1 expression level, tumor mutational burden (TMB), microsatellite instability (MSI), or mismatch-repair deficiency (dMMR) are not robust enough to predict adequate response for an individual patient. Whole transcriptomic datasets could provide more information on the level of an individual patient and could help to uncover biomarker candidates sufficiently robust for clinical application. Here, the goal of our study was to identify datasets where both gene expression data, treatment information, and clinical response data are simultaneously available. We evaluated only studies with publicly available data where no further action is needed for data acquisition.

Data collection and analysis was executed using steps recommended by the Preferred Reporting Items for Systematic reviews and Meta-Analyses (PRISMA) guidelines [52]. First, we searched the National Center for Biotechnology Information Gene Expression Omnibus (NCBI GEO) repository [53, 54] using the keyword “*human [organism] AND (pembrolizumab OR nivolumab OR atezolizumab OR durvalumab OR avelumab OR cemiplimab OR ipilimumab OR camrelizumab OR cintilimab OR tislelizumab OR toripalimab)”.* The search was performed multiple times with the last run on 22.10.2021. We also conducted another different search combination: *“human [organism] AND (anti-PD-1 OR anti PD-1 OR anti-PD-L1 OR anti PD-L1 OR anti-CTLA-4 OR anti CTLA-4)”*. Using these two approaches, 215 series files were identified and used for screening. In another portal, The Cancer Research Institute iAtlas (CRI iAtlas) (https://www.cri-iatlas.org/) [55], six datasets have been uploaded with clinical response and expression values. Third, a meta-analysis published by Litchfield et al. [56] was also used for data-searching, along with two similar analyses by Chen et al. [57] and Liu et al. [58]. From these three sources, 13 publications were investigated. Expression datasets were eligible for our review regardless of methods used for transcriptome analysis (e.g. RNA-sequencing or microarray), and data type (raw or processed).

**Fig. 2:**
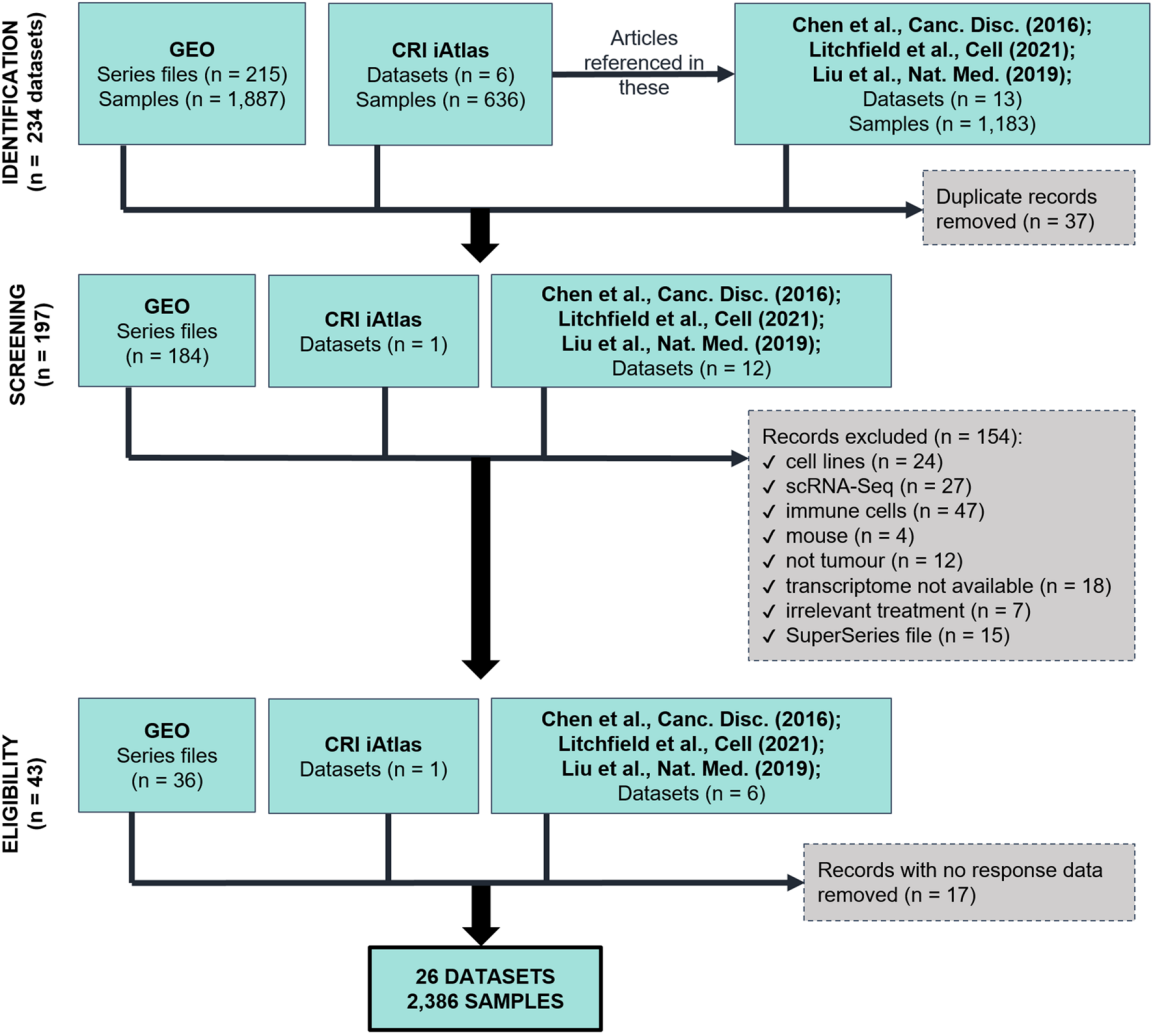
PRISMA flow diagram of data acquisition

Altogether, from NCBI GEO, CRI iAtlas, Litchfield et al., Chen et al., and Liu et al., 234 datasets have been found and investigated, out of which the duplicate records have been removed (Fig. 2). Out of eleven studies from Litchfield et al., four were also available on CRI iAtlas, and from these four, two (GSE78220, GSE91061) were also uploaded to NCBI GEO. Of the CRI iAtlas datasets, three (GSE121810, GSE91061, GSE78220) were also found in NCBI GEO. The Gide 2019 dataset was available only in CRI iAtlas. We have excluded datasets from (1) cell lines, including primary cell cultures established from biopsies, secondary cell lines, and stem cells, (2) single-cell RNA-sequencing (scRNA-Seq), including T cell or B cell receptor sequencing (TCR/BCR-Seq), and also if cell sorting was used and sequencing was conducted on a pre-defined, small amount of cells, (3) immune cells (e.g. T cells, DCs) or peripheral blood mononuclear cells (PBMC), (4) mice, (5) other diseases than cancer, (6) non-coding RNA profiling, methylation profiling (e.g. chromatin immunoprecipitation followed by sequencing (ChIP-Seq)), whole-exome sequencing (WES), protein array, or RNA expression data unavailability/inconsistency, (7) therapy other than immune-checkpoint inhibitors, and (8) GEO SuperSeries files. The filtering criteria aimed to involve only those studies where robust response and expression data were publicly available. Response data could either mean (1) progression-free survival (PFS) time, (2) overall survival (OS) time, (3) relapse-free survival (RFS) time, (4) progression-free interval (PFI) time, (5) recurrence, (6) response, and (7) response form (complete response (CR), partial response (PR), stable disease (SD), or progressive disease (PD) by the Response Evaluation Criteria in Solid Tumors (RECIST).

Finally, 26 datasets have met all eligibility criteria and were selected for our article; comprising 2386 samples from 1830 patients (Fig. 2). In these, gene expression was analyzed with distinct methods including microarrays (n = 4), quantitative real-time polymerase chain reaction (RT-qPCR) (n = 1), RNA-sequencing (RNA-Seq) (n = 15), and NanoString nCounter platforms (n = 6).

## Datasets with anti-CTLA-4 monotherapy

Three publications had samples treated solely with anti-CTLA-4 monotherapy. Tremelimumab was investigated in two different cohorts comprising a discovery dataset (phase III study) and a validation dataset (phase II study) set up to identify blood-based biomarkers [59]. The gene expression data from 360 patients include whole blood specimens analyzed before and after tremelimumab therapy (720 samples) and is available via the NCBI GEO ID GSE94873. In the original study, a gene expression-based classifier model was built to find genes response-predictive to anti-CTLA-4 blockade. The discovery dataset included 210 treatment-naïve metastatic melanoma patients with no prior chemotherapy and the validation dataset subsumed 150 chemotherapy-refractory melanoma patients. Both datasets consisted of pre-treatment blood samples, but only those patients were included in this study where post-treatment samples were also available. OS censoring (dead or alive) and response (responder or non-responder) designations have been uploaded to NCBI GEO. Response was determined by RECIST based on the patients’ radiological results. In both datasets, the objective response was only 13%, and because of progression, the one-year survival was higher in the pre-treatment discovery dataset (56%) than in the validation set (29%). 169 genes were measured with quantitative PCR. Nine predictors and six enhancers were identified from the tremelimumab pre-treatment expression profile that indicates an antitumor immune response. The enhancer variables do not have an indirect connection with the outcome but are highly correlated with predictor genes [60]. A classifier model using the expression of 15 genes from the pre-treatment samples reached AUC (area under cover) values of 0.86 and 0.68 for predicting one-year survival in the training and validation sets, respectively [59].

In the second study, the effects of ipilimumab were investigated in metastatic melanoma patients to find genes differentially expressed in glycolysis and to find intratumoral T cells associated with metabolic fitness regarding glycolytic capacity. From 21 participants, 22 samples (n = 7 before ipilimumab (pre-treatment) and n = 15 after ipilimumab (post-treatment)) have been used for RNA-Seq gene expression analysis via the Illumina HiSeq 2500 platform. Transcripts per million (TPM) normalized read counts were uploaded to NCBI GEO and are available using the accession number GSE165278 [61]. Patient characteristics and clinical data (OS time, response duration) can be found at (http://www.hammerlab.org/melanoma-reanalysis/). Clinical benefit was defined as PFS ≥ 24 weeks after treatment initiation. Six patients experienced benefits from the treatment and 15 did not [62]. Immune cell composition was investigated by CIBERSORT [63], which outputs the relative abundance of 22 immune cell types for each sample. Ipilimumab was found to promote immune cell infiltration and metabolic fitness in patients with melanoma [61].

In the third study, samples were taken from metastatic melanoma patients before ipilimumab monotherapy. Formalin-fixed, paraffin-embedded (FFPE) tumor samples (and matched germline DNA) of 110 patients were studied by WES and by Illumina HiSeq2500 platform, the dataset is referenced as “VanAllen2015” in Table 1. [64]. For expression analysis, only 42 patients had RNA expression data available (40 matched with WES). Whole transcriptome was analyzed in the tumor microenvironment. From the supplemental material of [56], 100 samples are available, of which 34 have both transcriptome data and clinical data. In addition, the expression and clinical data of 42 samples can be found in the CRI iAtlas portal as well. Clinical response was stratified based on RECIST 1.1. criteria where patients with clinical benefit experienced CR, PR, or SD with OS > 1 year (n = 27), while 73 patients had no clinical benefit (PD or SD with OS < 1 year). Ten patients achieved long-term survival (OS > 2 years) but showed early tumor progression (PFS < 6 months). Considering the RNA-Seq cohort, out of 42 participants, 14 patients were responders, 23 were non-responders, and five of them were categorized as long-term survivors. Granzyme a (GZMA), perforin 1 (PRF1), CTLA-4, and PD-L2 overexpression, and signatures connected to cytolytic activity, immune infiltration, neoantigen load, and overall mutational load had a significant correlation with clinical benefit in patients treated with ipilimumab [64].

**Table 1:** Summary of datasets discussed

Dataset ID	Patient count	Tumor	Drug applied	Sample acquisition	Outcome	PMID
GSE67501	11	Renal cell carcinoma	Nivolumab	Pre-treatment	Response form by RECIST and response	27491898
GSE78220	27	Melanoma	Pembrolizumab	Pre-treatment	OS time and response form by RECIST	26997480
GSE79691	1	Melanoma	Nivolumab	Post-treatment	PFS	28193624
GSE91061	65	Melanoma	Nivolumab	Pre-treatment or post-treatment	OS, response form by RECIST, and response	29033130
	58					
	56					
GSE93157	65	Head and neck squamous cell carcinoma	Nivolumab	Pre-treatment	PFS and response form by RECIST	28487385
		Non-squamous non-small cell lung cancer	Nivolumab or pembrolizumab	Pre-treatment	PFS and response form by RECIST	
		Skin cutaneous melanoma	Nivolumab or pembrolizumab	Pre-treatment	PFS and response form by RECIST	
		Squamous non-small cell lung cancer	Nivolumab or pembrolizumab	Pre-treatment	PFS and response form by RECIST	
GSE94873	360	Melanoma	Tremelimumab	Pre-treatment or post-treatment	OS and response	28807052
GSE111636	11	Bladder cancer	Pembrolizumab	Not specified	Response	-
GSE115821	8	Melanoma	Unknown	Pre-treatment or post-treatment	Response	30127394
GSE121810	29	Glioblastoma	Pembrolizumab	Pre-treatment or post-treatment	PFI, OS, response form by RECIST, and response	30742122
GSE122220	10	Melanoma	Anti-PD-1 and ipilimumab	Pre-treatment or post-treatment	Response form by RECIST	–
GSE123728	13	Melanoma	Pembrolizumab	Pre-treatment or post-treatment	Recurrence	30804515
GSE139050	6	HER2- breast cancer	Durvalumab	Pre-treatment	Response	33176887
GSE136961	21	Non-small-cell lung cancer	Unknown	Pre-treatment	PFS and OS	31959763
GSE140901	24	Hepatocellular carcinoma	Nivolumab and/or ipilimumab, or sabatolimab (MBG453) with spartalizumab (PDR001)	Pre-treatment	PFI, OS, response form by RECIST, and response	34414122
GSE165252	40	Esophageal adenocarcinoma	Atezolizumab	Pre-treatment or post-treatment	PFI, OS, and response	33504550
GSE165278	21	Melanoma	Ipilimumab	Pre-treatment or post-treatment	OS, response duration	33588426
GSE165745	24	Melanoma	Pembrolizumab or nivolumab	Pre-treatment	Response	33951424
GSE176307	89	Urothelial cancer	Pembrolizumab/nivolumab or avelumab/atezolizumab/durvalumab	Pre-treatment	PFS and response form by RECIST	34294892
GSE181815	10	Thymic carcinoma	Pembrolizumab	Pre-treatment	Response form by RECIST	34622229
GSE183924	37	Esophageal and gastroesophageal junction adenocarcinoma	Durvalumab	Pre-treatment	RFS	34604072
Chen, 2016	31	Melanoma	Pembrolizumab and/or ipilimumab	Pre-treatment and/or post-treatment	Response form by RECIST, and response	27301722
Cristescu, 2018	236	Bladder cancer	Pembrolizumab	Pre-treatment	Response	30309915
		Triple-negative breast cancer	Pembrolizumab	Pre-treatment	Response	
		ER + HER2-breast cancer	Pembrolizumab	Pre-treatment	Response	
		Colorectal adenocarcinoma	Pembrolizumab	Pre-treatment	Response	
		Head and neck squamous cell carcinoma	Pembrolizumab	Pre-treatment	Response	
		Melanoma	Pembrolizumab	Pre-treatment	Response	
		Small cell lung cancer	Pembrolizumab	Pre-treatment	Response	
Gide, 2019	74	Melanoma	Pembrolizumab and/or nivolumab and/or ipilimumab	Pre-treatment	PFI, OS, response form by RECIST, and response	30753825
Liu, 2019	121	Melanoma	Nivolumab or pembrolizumab	Pre-treatment	PFS, OS, response form by RECIST, and response	31792460
Mariathasan, 2018	348	Urothelial cancer	Atezolizumab	Pre-treatment	OS, response form by RECIST, and response	29443960
VanAllen, 2015	42	Melanoma	Ipilimumab	Pre-treatment	PFS, OS, response form by RECIST, and response	26359337

## Solid tumor datasets with anti-PD-1 monotherapy

With more than ten studies, datasets with anti-PD-1 monotherapy represent the largest groups of available transcriptomic studies. The most plausible reason for the popularity of anti-PD-1 monotherapy is the tumor origin-independent applicability of pembrolizumab Table 1 lists all datasets discussed in this chapter.

As previously discussed, PD-L1 expression is often used as a predictive biomarker for anti-PD-1 and anti-PD-L1 treatments. Yet, despite patient selection, response rates can still be very low. An earlier study [65] investigated the possible reasons behind failed anti-PD-1 (nivolumab) treatment in patients where high PD-L1 expression (≥ 5% of tumor cell surface staining) was observed by immunohistochemical staining (IHC). Pre-treatment FFPE samples of 13 renal cell carcinoma patients were analyzed by multiplex RT-qPCR and the Illumina Human HT-12 WG-DASL V4.0 R2 expression BeadChip and the expression data is available as GSE67501 in NCBI GEO. Patients were classified as responders or non-responders based on radiographic staging according to RECIST. 223 genes showed differential expressions when comparing responder and non-responder samples. Genes upregulated in non-responders were related to metabolic pathways and transport (e.g. UDP glucuronosyltransferase family 1 member a complex locus (UGT1A)), while genes upregulated in responders connected to immune functions. Patients who responded well to nivolumab overexpressed BTB domain and CNC homolog 2 (BACH2) and C–C motif chemokine ligand 3 (CCL3), genes important in initiating immune response [65].

Recurrent but surgically resectable glioblastoma patients were analyzed in another trial to compare survival benefit differences between neoadjuvant and adjuvant anti-PD-1 therapy. Transcriptome has been analyzed from 29 patients’ fresh frozen samples with Illumina HiSeq 3000 RNA sequencing and is available as GSE121810 [66]. Patients who received neoadjuvant pembrolizumab with adjuvant therapy had extended overall survival (median OS was 417 days) compared to those who received only post-surgical (adjuvant) anti-PD-1 therapy (median OS 228 days). Median PFS was 99.5 and 72.5 days in the neoadjuvant and adjuvant arms, respectively. Within the tumor, suppression of cell-cycle-related genes and blocked proliferation were observed with induction of PD-L1 in the tumor microenvironment. A link between tumor-infiltrating lymphocyte (TIL) density and survival was proposed [66].

Pre-treatment FFPE samples from metastatic NSCLC patients were analyzed to find gene expression signatures or single genes linked to response to anti-PD-1 therapy (nivolumab or pembrolizumab) in GSE136961. Sequencing was performed on an Ion S5™ XL Sequencer using an Ion 530 Chip with a 395 immune-related gene panel (Oncomine Immune Response Research Assay). PFS was longer in patients who had higher M1 macrophage- or peripheral T cell signature scores, these signatures performed best to discriminate between patients with or without durable clinical benefit. Longer PFS with durable clinical benefit is also associated with tumor necrosis factor receptor superfamily member 9 (TNFRSF9 or CD137) and proteasome 20S subunit beta 9 (PSMB9) overexpression [67]. CD137 plays a role in immune recognition and antitumor immune responses [68], while PSMB9 is involved in the immunoproteasome maturing [69]. M1 signature, peripheral T cell signature, CD137, and PSMB9 achieved better predictive performance than PD-L1 IHC, TMB, or the presence of TILs [67].

Metastatic urothelial (bladder, ureter/renal pelvis) cancer samples of 103 patients were analyzed in an investigation to find out whether fibroblast growth factor receptor 3 (FGFR3)-altered phenotype correlates with altered response to ICIs. This retrospective study involved patients treated with different ICIs, though all were administered as a monotherapy. 89 patients’ RNA from FFPE samples were sequenced using the Illumina NovaSeq6000 platform, of these patients, 47 had pembrolizumab, five had nivolumab, thirty-four had atezolizumab, two had durvalumab, and one had avelumab therapy. The majority of patients received prior chemotherapy as well. DNA sequencing was also conducted with a targeted mutation panel. Log2-transformed expression results have been uploaded to NCBI GEO and are available as GSE176307. The study demonstrated that FGFR-mutant and wild-type urothelial cancers are both sensitive to ICIs and have an equivalent T cell receptor diversity. A CD8 + T cell gene expression signature was found to be a good predictive biomarker [70].

Thymic carcinoma is a highly aggressive and rare malignant disease of the epithelial cells of the thymus. Thymic carcinoma patients who were treated with pembrolizumab and experienced recurrence after chemotherapy were evaluated in a phase II study (NCT02364076). The primary endpoint was one-year response rate, which reached 22.5% and the trial’s goal was to find molecular predictors of pembrolizumab responsiveness. Illumina HiSeq 4000 derived data from FFPE samples can be accessed as GSE181815. Among non-responders, PD-L1 and genes related to interferon-gamma (IFN-γ) response have been down-regulated, and M2 macrophages showed higher abundance [71].

A pan-cancer analysis published by Cristescu et al. in 2018 aimed to find universal predictive biomarkers for pembrolizumab monotherapy. The study included bladder cancer, triple-negative breast cancer, hormone receptor-positive HER2-negative breast cancer (estrogen-receptor-positive, Erb-B2 receptor tyrosine kinase 2-negative) colorectal adenocarcinoma, head and neck squamous cell carcinoma, melanoma, and small cell lung cancer samples [72]. T cell–inflamed gene expression profiles from 312 patients were determined by the NanoString nCounter platform. Correlations of TMB and gene expression profile (GEP) with best overall response (BOR) and PFS were studied in pre-treatment FFPE samples in patients who had both WES and expression data available. To have BOR, patients had to experience PR or CR. The authors claim that low levels of both TMB and T-cell inflamed GEP were tissue-agnostic factors and predict low response to anti-PD-1 therapy. The best response was seen in patients with high TMB and GEP, or high PD-L1 IHC expression and TMB [72]. WES data and clinical information about patients can be requested through the NCBI Database of Genotype and Phenotype (dbGAP) under the accession number phs001572.v1.p1.

Finally, in this chapter of studies, we also identified one GSE dataset where no publication is available (GSE111636). In this study, 11 patients with urothelial cancer (bladder cancer) were investigated who received pembrolizumab therapy until disease progression or for 2 years (termination of the study). FFPE samples were used on GeneChip Human Transcriptome array (HTA2.0) Arrays (Affymetrix, Santa Clara, CA) and the Robust Multichip Average (RMA) log2 signal intensity data was uploaded to NCBI GEO.

## Malignant melanoma datasets with anti-PD-1 monotherapy

All together seven studies used melanoma samples. In the first of these, pre-treatment metastatic melanoma biopsies were used for whole-exome sequencing (WES) and RNA-sequencing to identify a sensitivity signature for anti-PD-1 therapy (pembrolizumab or nivolumab). Illumina HiSeq2000 platform was used to determine gene expression in 28 samples and the data can be acquired as GSE78220 [73]. Expression values were analyzed by determining differentially expressed genes (DEGs) coupled with Gene Ontology (GO) enrichment and by differential signature enrichment based on single-sample gene set variance analysis (GSVA) scores. Twenty-six transcriptomic signatures were found to be co-enriched in the non-responder group, referred to as the IPRES signature (“innate anti-PD-1 resistance” as these tumors lack response to initial therapy). Among the 26 signatures, genes associated with the regulation of cell adhesion, extracellular matrix-remodeling, angiogenesis, wound healing, and epithelial-mesenchymal transition were overexpressed in the innately resistant tumors. Aldehyde dehydrogenase 1 family member L2 (ALDH1L2) and microfibril associated protein 2 (MFAP2) were the most significantly upregulated genes in non-responders, and cadherin 1 (CDH1) in responders. Responders were those patients who had CR, PR, or SD and non-responders had PD [73].

An alternative approach using post-mortem sample acquisition was executed to study factors associated with response to anti-PD-1 therapy in classical, high mutational burden cutaneous melanoma metastases [74]. Eight hundred twenty-seven genes were shown to be differentially expressed when comparing metastases that had regressed after anti-PD1 therapy to those that had progressed. In particular, laminin subunit alpha 3 (LAMA3), a gene involved in the formation of extracellular matrix and epithelial-mesenchymal transition, was found to be the most differentially expressed gene in the progressing metastases which was also confirmed at the protein level. Gene expression was measured with Illumina Human HT-12 WG-DASL V4.0 R2 expression BeadChip and the gene expression data for this cohort is available as GSE79691 [74].

Advanced melanoma samples of 68 nivolumab-treated patients were collected in the CA209-038 study [75]. Participants had either progressed on ipilimumab or were ipilimumab-naïve and did not receive chemotherapy. Response was determined by RECIST based on patients’ radiological results. For gene expression analysis, 65 patients’ 109 tissue samples were available (58 on-treatment and 51 pre-treatment) as GSE91061. The aim of RNA-seq in the article was to identify differentially expressed genes on-therapy between patients who experienced CR/PR and PD. Significantly overexpressed genes were linked to immune recognition, T cell activation, and lymphocyte aggregation. Most importantly, interleukin-17 receptor e (IL17RE), interleukin-17 receptor c (IL17RC), and FGFR3, all involved in the regulation of tumor microenvironment (TME), were found in this group [75].

Heterogeneous tumor population including pre-treatment skin cutaneous melanoma or melanoma (n = 25), head and neck cancer (n = 5), advanced non-squamous cell lung cancer (n = 22), and squamous cell lung cancer (n = 13) samples are available in the GSE93157 dataset [76]. There was no other treatment between biopsy and anti-PD-1 treatment initiation. The patients received either pembrolizumab or nivolumab (not both). For gene expression analysis, FFPE samples were used in the PanCancer 730 Immune Panel on the nCounter system. The study identified 23 immune-related genes in connection with response and PFS. The authors’ investigations showed that PD-1 gene expression and 12 signatures connected to T-cell, and NK cell activation were associated with non-progressive disease and better PFS regardless of tumor type, treatment, or biopsy time [76].

Recently, Liu et al. published a large study with 144 metastatic melanoma patients treated with anti-PD-1 (85 pembrolizumab,- and 59 nivolumab-treated), either as a first (n = 71),- or a second-line therapy (n = 73) [58]. Sixty of them also received prior anti-CTLA-4 blockade (ipilimumab). Transcriptome analysis with Illumina HiSeq 2000 v.3 or HiSeq 2500 platforms was executed for 121 patients and the raw RNA expression data can be found in dbGaP under the accession number phs000452.v3.p1. TPM-normalized expression values and clinical response, PFS, and OS are provided by the authors as supplementary material. Best overall response was calculated according to RECIST 1.1. While TMB as a predictive biomarker varied between melanoma subtypes, MHC-I,- and MHC-II-associated genomic and transcriptomic features had a better correlation with response. All 13 of MHC-II class,- and the majority of MHC-I-associated genes were overexpressed in responders [58].

We have to note here two smaller studies with metastatic melanoma samples which were published under the NCBI GEO accession numbers GSE123728 [77] and GSE165745 [78] with 13 and 24 patients, respectively. Both studies administered anti-PD-1 monotherapy (pembrolizumab or nivolumab) and determined gene expression using the NanoString nCounter platform from FFPE samples.

## Datasets with anti-PD-L1 monotherapy

Anti-PD-L1 monotherapy data was available in five studies, each examining a different solid tumor type. The largest of these investigated the effects of atezolizumab in 429 pre-treatment metastatic urothelial cancer (mUC) patients samples from a phase II clinical trial (IMvigor210, with 310 participants from NCT02108652, and 119 participants from NCT02951767) [79]. The trial endpoint was calculated from objective response rates. WES and RNA-Seq have been conducted on FFPE samples, for 250 and 368 patients, respectively. RNA-Seq and WES data, along with patient clinical characteristics have been deposited to the European Genome-Phenome Archive under accession number EGAS00001002556. 272 patients received previous platinum-based chemotherapy. The study found that PD-L1 expression on immune cells but not on tumor cells was associated with improved response (suggesting a pre-existing T cell immunity). Overexpression of interferon-gamma receptor 1 (IFNGR1), transforming growth factor beta 1 (TGFB1), and transforming growth factor beta receptor 2 (TGFBR2) showed higher expression in non-responders and were correlated to reduced OS. IFNGR1 mediates adaptive resistance to checkpoint inhibitors, while TGFB1 and TGFBR2 have distinct pro-tumorigenic and anti-immunogenic roles in human cancers [79].

Another study explored hepatocellular carcinoma (HCC) in 42 patients who received either nivolumab monotherapy or anti-PD-1 (pembrolizumab) in combination with anti-CTLA-4 (ipilimumab). FFPE samples of 24 patients were analyzed with NanoString nCounter PanCancer Immune Profiling for 770 genes. Processed and raw data, along with clinical data have been uploaded to NCBI GEO as GSE140901. Subjects who had objective responses had higher expression of genes related to T cell exhaustion. Between responders, nine genes were found to be overexpressed and were able to predict PFS and OS for metastatic HCC patients [80].

Two, phase II studies focused on the upper gastrointestinal application of anti-PD-L1 monotherapy including atezolizumab therapy in esophageal carcinoma (PERFECT) and durvalumab therapy in esophageal and gastroesophageal junction adenocarcinoma (NCT02639065). In PERFECT, Illumina HiSeq 4000 RNA sequencing was used for gene expression analysis of 77 endoscopic biopsy or resection tumor samples (GSE165252). Samples were acquired at three different time points (1) before treatment (called “baseline”) (2), on-treatment (3rd week), and (3) in case a poor response was suspected from resection sample (called “resection”). In this study, only 10 patients had CR. Overexpression of a 6-gene IFN-γ signature differentiated responders at the baseline. The authors concluded that combining neoadjuvant chemoradiotherapy with atezolizumab was feasible in patients with esophageal carcinoma [81]. In NCT02639065, FFPE samples were used in the Illumina NovaSeq 6000 platform, the primary endpoint was 1 year relapse-free survival (RFS), and 37 patients’ normalized expression data (fragments per kilo base of transcript per million mapped fragments (FPKM)) has been uploaded to NCBI GEO (GSE183924), along with RFS time. Adjuvant durvalumab therapy caused improvement in one-year RFS and was associated with the presence of M2 tumor-associated macrophages (TAMs), along with memory T cells [82].

Lastly, a small study with six patients (3 responders and 3 non-responders) from the NCT02802098 trial investigated advanced HER2-negative breast cancer to find immuno-priming benefits of bevacizumab before anti-PD-L1 treatment (durvalumab). FFPE samples were sequenced on an Illumina HiSeq 2500 platform and the normalized RNA-Seq read counts were uploaded to NCBI GEO as GSE139050 [83].

## Datasets with anti-PD-1 and anti-CTLA-4 combination therapy

Investigation of combination therapy has already been mentioned for some patients in the above-described studies. These regimens are generally rare and only three published studies and one yet to be published study focused on such patients. All these studies investigate metastatic melanoma patients.

The largest number of specimens were evaluated in the most recent study published by Gide et al. in 2019 [84]. In their study involving 120 patients, immune profiles were correlated with response to anti-PD-1 (pembrolizumab, nivolumab) monotherapy (n = 63) or anti-PD-1 and anti-CTLA-4 (ipilimumab) combination therapy (n = 57). Besides Illumina HiSeq 2500 RNA sequencing, clinical data including treatment, sex, RECIST response, PFS, OS, and time of sample acquisition were provided by the authors. Responders were defined by RECIST 1.1 and non-responders were defined as those with a PD or SD ≤ 6 months before progression. RNA-Seq data were deposited in the European Nucleotide Archive (ENA) (PRJEB23709). The study identified eomesodermin-positive, cluster of differentiation 69,- and 45 RO-positive (EOMES + CD69 + CD45RO +) effector memory T cells to be associated with better response, longer PFS, and tumor shrinkage [84].

Another study developed a new algorithm dubbed IMPRES (IMmuno-PREdictive Score), which can predict response to anti-PD-1 and/or anti-CTLA-4 immune-checkpoint inhibitors in metastatic melanoma. The prediction itself is based on pairwise relations between 28 immune checkpoint genes’ expression data with known co-inhibitory or co-stimulatory effects. IMPRES correctly identified all true responders while misclassified less than half of the non-responders. Overall, it achieved an of AUC = 0.83 for accuracy. RNA was purified from patients’ frozen or FFPE specimens and gene expression was measured with the Illumina HiSeq 2000 or Illumina NextSeq 500 RNA-sequencing platforms from 37 samples in total (GSE115821) [85]. The work identified that cluster of differentiation 27 and 40 (CD27, CD40), and herpes virus entry mediator (HVEM) gene expression is correlated to a better response to immune checkpoint blockade. CD27 and CD40 play a key role in activating T cells and anti-tumor immune responses [86–88], while HVEM is involved in both activating and inhibiting it [89]. The study also showed the expression of other genes such as the cluster of differentiation 200 and 276 (CD200, CD276/B7-H3), T-cell immunoglobulin domain and mucin domain 3 (TIM-3), and v-domain immunoglobulin suppressor of T cell activation (VISTA) correlated with a worse response [85]. CD200, CD276, TIM-3, and VISTA regulate immunosuppression, resulting in an inhibitory checkpoint signal [90–94].

In a further project, 53 patients with metastatic melanoma were treated with sequential anti-CTLA-4 (ipilimumab) and anti-PD-1 (pembrolizumab) therapy. CTLA-4 blockade was induced first, and in the case of progression, it was continued with PD-1 blockade. Of the 53 patients, 46 progressed after anti-CTLA-4, and from these, 13 responded to anti-PD-1 therapy. A separate patient cohort was also included with 16 anti-CTLA-4-naïve patients, who received only anti-PD-1 therapy. Patients were stratified as responders if radiographic images displayed no evidence of disease or having SD or reduced tumor size for > 6 months. Immune profiling of 795 genes was executed with the NanoString nCounter platform. The authors concluded that immune signatures should be evaluated shortly after starting the treatment (rather than pre-treatment) because this timing was found to be more a robust predictor of response to ICI. Differential effects of anti-CTLA-4 and anti-PD-1 to TME have been also found, along with potential resistance mechanisms to ICIs [57]. Results of the NanoString expression analysis from this patient cohort have also been re-analyzed in [95].

To end, we can mention here one small dataset from NCBI GEO (GSE122220) with only ten patients and without a publication. The deposited metastatic melanoma tumor biopsies were analyzed by Illumina HumanHT-12 V4.0 expression beadchips.

## Animal studies

Considering the difficulties of using cell lines in animals, and the indirect representativeness of these models to tumors with intact humane immune systems, it is no wonder that only a few animal studies are available. Nevertheless, some aspects of molecular oncology can only be studied in animals and for this reason, we briefly summarize below transcriptomic datasets stemming from mice studies (see also Fig. 2).

In GSE129127, a cohort of 95 melanoma patient-derived xenograft (PDX) samples in NOD.Cg-Prkdcscid Il2rgtm1Wjl/SzJ host mouse was analyzed with the Illumina HiSeq 2000 platform. Tumor fragments from melanoma metastases were injected subcutaneously and this study aimed to compare “stromal immune” (SIM) and “tumor-autonomous inflammation” (TAF) signatures based on expression data. The SIM signature was associated with response to anti-CTLA-4 therapy only, and the TAF signature predicted response to anti-PD-1 only. Interestingly, when used together, these two signatures also predicted response to combination therapy [96].

A second study of 21 samples from a C57BL/6 mouse strain with B16F10-Alkbh5 KO, or B16F10-Fto KO implanted tumors, in combination with a B16F10-NTC control was analyzed with Illumina HiSeq 4000 platform after immunotherapy [97]. One patient with metastatic melanoma who has been treated with anti-PD-1 therapy also provided a sample for scRNA-Seq. RNA-Seq along with m6A RNA immunoprecipitation followed by high-throughput sequencing (MeRIP-Seq) was used to investigate if gene expression and regulatory changes are a consequence of Alkbh5 or Fto-mediated m6A/m6Am demethylation. The transcriptomic data from this study is available to download as GSE134388.

Two smaller additional studies with less than twenty specimens are available. 19 samples from wild-type and lysine demethylase 1b,- or 1a-knockout (KDM1BKO or KDM1AKO) immunocompetent Balb/cJ mice and athymic BALB/c nude mice were analyzed after subcutaneous injection with MDA-MB-231 breast cancer cells in GSE135400. WT and KO cell lines were also sequenced in a HiSeq X Ten platform. KDM1B was found to be a key component in response, but the complete results are still unpublished. Eight samples (two control and six treated) from metastatic melanoma-bearing humanized mice after anti-PD-1 therapy analyzed with Illumina NextSeq 500 were published as GSE161351. Normalized RNA-seq data was used to enumerate tumor-infiltrating leukocytes using CIBERSORT and the study concluded that mast cells are associated with ICI resistance [98].

## Conclusions

In this review, we summarized datasets with available transcriptomic and clinical response data from patients treated with immune-checkpoint inhibitors. The review was set up to group available datasets based on the investigated treatment. In addition, we also summarized the most important results of the individual datasets.

Of note, there are other options for immunotherapy besides immune-checkpoint inhibitors. Cancer vaccines (e.g. sipuleucel-T), oncolytic viruses (talimogene laherparepvec), other immunomodulators, adoptive cellular immunotherapy, chimeric antigen receptor T-cell (CAR-T) immunotherapy (e.g. tisagenlecleucel), or NK cell therapy can also be administered to patients. However, the discussion of these was out of the scope of the current review.

There is an important limitation of our review. as ongoing clinical trials about the new generations of ICIs (e.g. targeting TIM-3, CD223/LAG-3, CD276/B7-H3, B7-H4, A2aR, CD73, CD94/NKG2A, PVRIG/PVRL2) were not included due of the lack of linked transcriptomic datasets. An exception for this was GSE140901, where sabatolimab (MBG453), targeting TIM-3, was analyzed—but this study also involved anti-PD-1 and anti-CTLA-4 therapies. TIM-3 is an inhibitory receptor on T cells and is usually co-expressed with other immune checkpoint receptors. The feasibility of TIM-3-targeting drugs in both solid, and hematological tumors is being tested in phase I and II studies either as a monotherapy or a combination therapy (e.g. with anti-PD-1) [99]. Another prominent candidate is lymphocyte activation gene 3 (LAG-3) or CD223, which can be found on the surface of many immune cells mediating antitumor-immunity [100]. The efficacy of LAG-3-targeting is under investigation in phase I and II clinical trials in a wide variety of cancers [101]. B7-H3 or CD276 is expressed on APCs and plays a dual role in the immune system, as it can also facilitate co-inhibitory and co-stimulatory signals. Targeting of B7-H3 (e.g. with mAbs or antibody–drug conjugates) is being studied in phase I-III trials [90]. Another member of the B7 family, B7-H4, is also investigated in phase I and II clinical trials. B7-H4 is involved in the inhibition of immune response and can be found on APCs and tumor cells [102]. Adenosine is overproduced in the tumor microenvironment and upon binding to its receptor on immune cells, adenosine 2A receptor (A2aR) mediates an immunosuppressive signal [103]. Antagonists of A2aR or blocking agents of the adenosine production itself via targeting CD73 are currently in phase I and II studies either as monotherapies or combination therapies [104]. Natural Killer Group Protein 2 (NKG2A) or CD94 is expressed on NK cells and CD8 + T cells in the TME, contributing to a failed immune recognition. An NKG2A-targeting antibody, monalizumab, is currently investigated in phase I and II studies with different study designs [105]. Poliovirus receptor-related immunoglobulin domain-containing protein (PVRIG) or CD112R and poliovirus receptor-related protein 2 (PVRL2), or CD112 or nectin-2 are also promising therapeutic targets. PVRIG is a co-inhibitory receptor of the DNAM/TIGIT/CD96 family and binds to PVRL2, both abnormally expressed in human cancers [106].

We have also omitted studies where clinical or expression data had to be acquired from drug companies or other websites, or simply were not available. For example, in the Snyder et al. 2017 PLoS Medicine paper, the availability of expression data is not mentioned by the authors [107]. Likewise, in the Snyder et al. 2014 NEJM paper 19 samples were used for transcriptome analysis but the original article [108] didn’t mention any expression analysis. Another study by McDermott et al. published in Nature Medicine in 2018 used 48 samples for transcriptome analysis, and access to the expression data might be requested by the accession number EGAS00001002928 [109].

The number of retrospective studies investigating predictive biomarkers useful for immune checkpoint inhibitors is still low. Our review was set up to enable the reader to be acquainted with transcriptome-level datasets while maintaining a bird’s eye view of the entire field. Selection and combination of the most relevant datasets will enable rapid independent validation of future biomarker candidates correlated to ICI therapy response.

## Data Availability

Data sharing is not applicable to this article as no datasets were generated or analysed during the current study.
